# Pediatric anesthesia and achalasia: 10 years’ experience in peroral endoscopy myotomy management

**DOI:** 10.1186/s44158-022-00054-7

**Published:** 2022-06-13

**Authors:** Fabio Sbaraglia, Pietro Familiari, Federica Maiellare, Marco Mecarello, Annamaria Scarano, Demetrio Del Prete, Rosa Lamacchia, Federica Antonicelli, Marco Rossi

**Affiliations:** 1 Department of Anesthesia and Intensive Care, Fondazione Policlinico Universitario “A Gemelli” IRCCS, Roma, Italy; 2 Digestive Endoscopy Unit, Fondazione Policlinico Universitario “A Gemelli” IRCCS, Roma, Italy

**Keywords:** Pediatric anesthesia, Endoscopy, Adverse events, Rapid sequence induction, Mechanical ventilation

## Abstract

**Background:**

Endoscopic treatment for achalasia (POEM) is a recently introduced technique that incorporates the concepts of natural orifice transluminal surgery. Although pediatric achalasia is rare, POEM has been episodically used in children since 2012. Despite this procedure entails many implications for airway management and mechanical ventilation, evidences about anesthesiologic management are very poor. We conducted this retrospective study to pay attention on the clinical challenge for pediatric anesthesiologists. We put special emphasis on the risk in intubation maneuvers and in ventilation settings.

**Results:**

We retrieved data on children 18 years old and younger who underwent POEM in a single tertiary referral endoscopic center between 2012 and 2021. Demographics, clinical history, fasting status, anesthesia induction, airway management, anesthesia maintenance, timing of anesthesia and procedure, PONV, and pain treatment and adverse events were retrieved from the original database.

Thirty-one patients (3–18 years) undergoing POEM for achalasia were analyzed. In 30 of the 31 patients, rapid sequence induction was performed. All patients manifested consequences of endoscopic CO_2_ insufflation and most of them required a new ventilator approach. No life-threatening adverse events have been detected.

**Conclusions:**

POEM procedure seems to be characterized by a low-risk profile, but specials precaution must be taken. The inhalation risk is actually due to the high rate of full esophagus patients, even if the Rapid Sequence Induction was effective in preventing ab ingestis pneumonia. Mechanical ventilation may be difficult during the tunnelization step. Future prospective trials will be necessary to individuate the better choices in such a special setting.

## Introduction

Peroral endoscopy myotomy (POEM) is a new endoscopic technique for the treatment of achalasia, which encompasses the concepts of natural orifice transluminal endoscopic surgery [1].

Since the first human case was performed in Japan in 2008 [2], this procedure has been successfully tested on more than 5000 adult patients nowadays. Although pediatric achalasia is rare, POEM has also been successfully used for the treatment of symptomatic children since 2012 [3].

POEM combines the benefits of a minimally invasive, endoscopic, transoral procedure, with the long-term efficacy of a surgical myotomy, so for achalasia disorder, it has emerged as a first-line treatment [4]. The procedure includes incision of the esophageal mucosa, dissection of the submucosa facilitated by insufflation of carbon dioxide (CO_2_), and myotomy of the esophageal muscular layer and requires special and often challenging anesthesiological management [5–7].

Esophageal clearance is severely compromised, and frequently, at the time of surgery, esophagus is still full of food. Furthermore, during the procedure, large volumes of carbon dioxide are inflated through the submucosal space into the mediastinum and the abdominal cavity, with possibly severe impairment of ventilation due to subcutaneous emphysema, capnomediastinum, capnoperitoneum, and pneumothorax [8].

Some studies about anesthesiological management are extrapolated from adult literature, while in the pediatric field the evidence is very poor, existing with only a few reports, focused on surgical procedures.

We perform a retrospective chart review about the anesthesiological management of 31 children who underwent POEM in a single center: we underline the clinical challenge in the perioperative period focusing on the risk in intubation maneuvers and ventilation management.

## Materials and methods

This retrospective study was approved by local IRB (prot.5486/14). All the patients underwent POEM at a single tertiary referral endoscopic center between January 2012 and December 2021. They were retrospectively identified from a prospectively collected database. Patients, who were younger than 18 years old at the time of the procedure, were included in this study.

Demographics, clinical history, fasting status, anesthesia induction, airway management, anesthesia maintenance, the timing of anesthesia and procedure, post-operative nausea and vomiting (PONV), pain treatment, and adverse events were retrieved from the original database and medical charts.

Data were collected in a prospective database (Microsoft Excel) and password protected. Parametric data were presented as mean data and range. Based on the small size of our sample, and on the descriptive presentation of data, statistical analysis for determination of risk odd ratio for each factor was not allowed. The study was conducted following the ethical principles outlined in the Declaration of Helsinki.

## Results

We collected a total of 31 patients. The firsts 6 procedures were scheduled in the operating room, but since 2013 POEM was performed exclusively in an endoscopic suite.

Preclinical assessment at the hospitalization the day before the procedure is summarized in Table 1. American Society of Anesthesiologists (ASA) class was assessed as II in 25 patients for the only presence of achalasia and nutritional status, as III in 6 patients for other pathologies (1 Down Syndrome affected by major heart disease; 1 hemiplegia due to oncologic neurosurgery; 4 chronic pulmonary disease, related with esophageal reflux). Preoperative preparation in the ward was standardized, similarly to our adult patients [9].

**Table 1:** Demographic and procedural data

31 patients data		
	Number	Range
M/F	*18/13*	
Age (years)	*11.8*	*(3–18)*
Weight (kg)	*39.1*	*(11–75)*
Height (cm)	*154.6*	*(95–183)*
ASA III/II (excepted for malnutrition)	*6/25*	
Timing of surgery (min)^a^	*49.3*	*(14–82)*
Timing of anesthesia (min)^a^	*109.6*	*(60–165)*
Timing of discharge (min)^a^	*66*	*(48–90)*

^a^This value excluded 2 procedures aborted before conclusions due to surgical issue

After two days of a clear liquid diet, the 24 h before the procedure adult patients could take just water, while an intravenous glucose solution assured the caloric intake in smaller patients (10/31, 32%).

On the day of the procedure, the antibiotic prophylaxis with clindamycin 20 mg/kg (600 mg maximum) and Cefazolin 30 mg/kg (2 g maximum) was administered iv 30 min before the beginning.

As in adults, a precautionary nasogastric tube was not placed before the procedure, except in one child, but no esophagus content was found.

Anesthesia induction was performed by intravenous drugs in all patients: in one case the anesthesiologist performed a standard induction with propofol, cis-atracurium, and fentanyl; in the other cases a rapid sequence induction (RSI), administering propofol (30/30), rocuronium (26/30) or succinylcholine (4/30) and fentanyl (15/30), remifentanil (4/30) or both (11/30), was preferred. Intubation was easily performed (Cormack 1 was reported in all patients) and no episode of ab ingestis pneumonia occurred. However, in 4 patients (12.9%), the laryngoscopy revealed the presence of some residue in the pharynx, and in 15 patients (48.4%), the endoscopy reported food debris.

General anesthesia was maintained with sevoflurane (25/31, 80.64%), desflurane (5/31, 16.1%), or propofol-target controlled infusion (1/31, 3.2%). Mechanical ventilation setup was volume controlled (VCV) (24/31, 77.42%) and pressure controlled (PCV) (7/31, 22.58%). In all patients, a standard positive end-expiratory pressure (PEEP) of 4–5 cm H_2_O was ensured.

In addition to opiates chosen for the induction, analgesia was improved by acetaminophen (31/31, 100%) and tramadol (12/31, 38.7%), while the PONV prophylaxis was ensured with iv dexamethasone before induction.

Two procedure was aborted because of technical difficulties in submucosal tunnelization, due to fibrosis. There was no anesthesiological indication to discontinue the procedure. However, many cases required adjustments in ventilation, because of an increase in peak inspiratory pressure (maximum value reported was 32 mmHg over the PEEP) or in EtCO_2_ values (maximum value reported was 60 mmHg) during the submucosal tunneling.

All patients manifested some transient signs of endoscopic CO_2_ insufflation (pneumomediastinum, pneumoperitoneum, and subcutaneous emphysema) but no one required any clinical therapy. Neither tension pneumothorax nor other life-threatening events were reported.

The mean time of surgery was 49.3 +/− 17 min, with an extubation time of 11 +/− 4 min. Once extubated all patients were observed in the recovery room for a mean period of 66 +/− 24 min. Mild analgesic rescue (tramadol) was administered in 7 patients (22.58%), while 1 received a stronger opiate (3.22%) (morphine). PONV occurred in 5 patients (16.13%), but it was effectively treated with ondansetron. No emergence agitation was reported. One case of transient bronchospasm without desaturation occurred in the recovery room, but therapy with salbutamol puffs and betamethasone iv quickly restored the normal pulmonary function.

All patients were regularly transferred to the ward and started feeding within 48 h, with discharge scheduled for the second postoperative day. A mild case of ab ingestis occurred in a 3-year-old baby in the second postoperative day, delaying discharge to the 8th day.

## Discussion

POEM procedure requires a specific anesthesiologic approach concerning strategy, risk, and possible adverse events. In 2014, Tanaka et al. made suggestions for adults [10], even though the first prospective study dates from 2015 [5], while in children the experience with this new procedure is limited (Table 2).

**Table 2:** Literature data about POEM in pediatric population

	Pts	Mean age (years)	Symptoms duration (months)	Timing of procedure (min)	Procedural adverse events	Occupant days
Maselli R *Endoscopy* [3]	1	3	No data	190	None	No data
Familiari P *J Ped Gastr Nut* [11]	3	9.5 (9–11)	17(3–36)	60.6 (49–76)	1 Mucosal Perforation	5 (4–7)
Li C *J Ped Surg* [12]	9	14.1 (10–17)	26.4 (6–60)	56.7 (45–105)	1 esophagitis 1 subcutaneous emphysema	No data
Tang X *Pediatr Surg Int* [13]	5	14.7 (12–17)	6.3 (1.5–12)	56 (40–90)	None	7 (5–13)
Caldaro T *J Pediatr Surg* [14]	9	12.2 (6–17)	No data	62 (49–74)	1 pneumoperitoneum 1 perforation 1 GERD	4.1 (2–7)
Chen W *Gatroint End* [15]	27	13.8 (6–17)	17 (0.2–36)	39.4 (21–90)	1 pneumothorax 2 pneumoperitoneum 5 mucosal perforation 1 fever 2 severe pain 4 GERD	3.2 (1–7)
Filser J *Eur J Ped Surg Rep* [16]	1	10	18	93	None	3
Petrosyan *J Pediatr Surg* [17]	2	No data	No data	No data	1 pneumothorax 1 GERD	No data

*GERD* Gastroesophageal reflux disease

This procedure has been seldom implemented in young patients for prudential reasons, but the good outcomes experienced in adult patients have led endoscopists to apply POEM also to younger patients.

The first point to be assessed is the risk of regurgitation at induction of anesthesia. To avoid aspiration literature suggests a pre-surgery liquid diet, withholding oral intake 24 h before, antacid therapy, and the use of rapid sequence induction [8]. Despite the fasting, in all patients with achalasia, residual food debris should always be suspected. Indeed, in our series, only 52% of patients had the esophagus completely empty at the beginning of the procedure.

Moreover, in children, prolonged fasting can lead to ketoacidosis and hypoglycemia [18], further worsening a nutritional status already debilitated by previous dysphagia [19]. Right now, there is no evidence that this approach improves esophageal clearance.

Although medical therapy has been demonstrated efficient in the reduction of lower esophageal sphincter (LES) pressure [20, 21], children were never premedicated. A pre-procedural treatment with nifedipine [22] or nitrates [23] could be useful in emptying improvement, but there is evidence neither on this approach.

In some centers, an active draining of esophageal content is performed in children as well as in adult patients. In our series, only one anesthesiologist chose to place preoperatively an orogastric tube. In all other cases (30/31), the performer deemed that the risk of the tube positioning outweighed the benefits. Indeed, the esophageal content could be either liquid (i.e., secretion, saliva) or solid (food debris), so no withdrawable. On the contrary, it should be taken into account the real risk of esophageal perforation [24], also considering the esophageal muscular wall alterations.

Performing an esophagoscopy for evacuation of solid esophageal contents within a few hours before POEM is suggested in adults [8–10]. Nabi et al. described a pre-operative endoscopy in children under light sedation [25], but the poor compliance of pediatric patients would require higher doses of sedative drugs, with a bigger risk for airway protection. Furthermore, the traditional fear of inhalation at induction must be reconsidered. Warner et al. evaluated pulmonary aspiration of gastric contents during the perioperative period in infants and children in 63.180 general anesthesia. Among them, only 24 children inhaled (0.04%) and only 3 patients required mechanical ventilation for more than 48 h [26].

At odds with the previous data, there is even more evidence that the traditional fear of inhalation of food debris, could be disproportionate. Some authors questioned even the rapid sequence induction for the high risk of vomiting in children [27]. The more recent review by Engelhardt et al. confirms the meager number of pulmonary aspirations, as well as highlights the risk linked to hypoxia and traumatic tracheal intubation when this technique is transferred directly into pediatric anesthesia practice [28].

The attitude of our group was more traditional, usually (30/31) preferring the RSI. No complication has been observed, even in smaller children. Sellick maneuver (cricoid pressure to occlude the esophagus during induction) was not performed because, despite current adult guidelines, studies on the pediatric population do not seem to show benefit in preventing regurgitation and potential risk in the airway management [29].

Some considerations are worth to be done about the ventilatory approach. Throughout the entire procedure, a continuous insufflation is performed to achieve a good display of the endoscopic field. Especially during the tunnelization step, when the esophageal wall is dissected, the gas insufflated cross in the points of least resistance, producing pneumediastinum, pneumoperitoneum, and subcutaneous emphysema. Since children’s anatomy is characterized by a smaller abdominal/thoracic cavity, a minimal increase in abdominal pressures could compromise diaphragmatic breathing [30].

In our series, all patients needed some revisions in the ventilator setting regardless of whether volume-controlled ventilation or pressure-controlled ventilation had been applied.

The better mechanical ventilation strategy for a normal pediatric lung is not still defined. Many papers only recognized the pathologic status [31] and most of them were published more than two decades ago [32]. Some indications can be obtained by authors who analyzed the patient’s outcome after several changes in mechanical ventilatory approaches [33], but as for pediatric mechanical ventilation, there is not any scientific evidence, so the anesthesiological management must be driven by personal experience and data from adults [34].

This lack of evidence appears in the extreme variability of the mechanical ventilation approach in our sample, especially when the anesthesiologist has to consider the continuous CO_2_ insufflation generated by the endoscope. At the beginning of the procedure, VCV was the preferred approach in most of the cases (24/31) and a minimal PEEP (4–5 cm H2O) was ensured in all patients.

Based on the data from the anesthetic chart, the entire sample needed some adjustment in ventilation parameters during the tunnelization and the myotomy. This appears as the most difficult step in ventilation control, due to some alterations in the compliance of the respiratory system. In adults, Tanaka et al. observed an increase of EtCO_2_ in all patients (until 50 mmHg) but no elevation of inspiratory pressure during mechanical ventilation was reported [10].

**Fig. 1:**
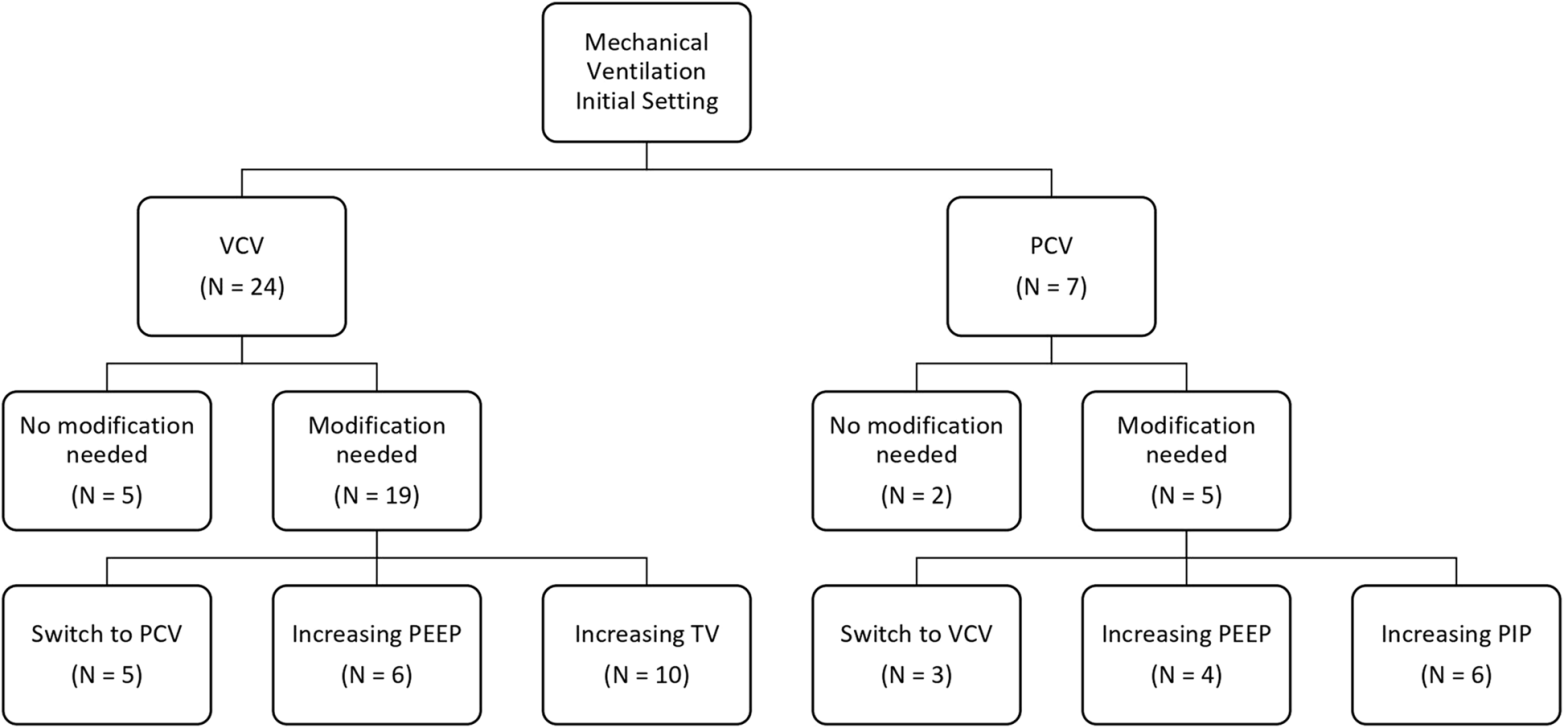
Modification in mechanical ventilation setting

In our series both parameters were compromised, suggesting that the differences existing in the pediatric chest wall, compared with adults, [35] were involved. Clinical treatment is extremely variable as results in Fig. 1. Despite this “Brownian motion” profile, no adverse events occurred, suggesting that the ventilator mode used does not have a strong impact on the respiratory adverse events. It is also worth noting that Plateau Pressure (Pplat) was never reported in clinical charts. Although a value above 35 cm H_2_O in adults is strongly correlated with barotrauma [36], no anesthesiologist used Pplat to guide mechanical ventilation.

Some authors tried to evaluate whether the pneumomediastinum and pneumoperitoneum occur using the postoperative CT scan. Abnormal findings were frequently identified on radiological examination, but no correlation was found with the development of complications [37]. Hence, none of these conditions have any real clinical impact on the outcome.

Indeed, at our center, it is not common to perform some types of radiological exams in the postoperative period, especially in pediatric patients.

According to the limited duration of the POEM procedure, the management of analgesia involves the use of short-acting drugs. During the stay in the Recovery Room (mean time 66 min), only 25.8% (8/31) patients required an analgesic rescue (7 with weak opioids and 1 with strong opioids), while 16.1% (5/19?) required an intravenous therapy for PONV.

No serious or life-threatening adverse events have been reported. Only two major complications (supposedly linked to achalasia) occurred, but without sequelae. The length of hospitalization (3 days for each patient) was coherent with literature data, which show a period between 1 and 5 days for the uncomplicated procedures [38].

Considering the level of evidence in our retrospective analysis, the POEM procedure seems to be characterized by a low-risk profile, also in children and adolescents. No serious adverse events occurred and the anesthesiological requirements are within the reach of most of the pediatric anesthesiologists. Thanks to a proper scheduling system in a well-equipped endoscopic suite, instead of an operating room, any difficulties were encountered. Moreover, the execution of the procedure in a more friendly environment is probably positive in reducing the psychological impact on children and their parents.

About anesthesiological issues, data that emerged from our case series are quite comforting. The inhalation risk is due to the high rate of full esophagus patients, but the RSI was effective in preventing ab ingestis pneumonia.

The mechanical ventilation may be difficult during the tunnelization step, but in any case, the procedure had to be delayed or stopped. According to literature, there is no recommendation on optimal intraoperative ventilation strategy. The set tidal volume or inspiratory pressures need to be changed according to EtCO2 and Pplat, while a certain level of PEEP in the range of 4–8 cmH_2_0 is necessary to prevent atelectasis.

Our experience would like to help fill the gap in pediatric management, reporting a significant case series, even considering the rarity of achalasia, mostly in children. However, considering the lack of evidence and the limited sample, we cannot make general recommendations. Future prospective trials are necessary to find better options in a such special setting.

## Data Availability

All data are available under request to the corresponding author.

## Abbreviations

ASA: American Society of Anesthesiology
CO_2_: Carbon dioxide
EtCO2: End-tidal CO_2_
GERD: Gastroesophageal reflux disease
PCV: Pressure controlled ventilation
PEEP: Positive end-expiratory pressure
POEM: Peroral endoscopy myotomy
PONV: Post-operative nausea and vomiting
RSI: Rapid sequence induction
SD: Standard deviation
